# The voltage-gated sodium channel EF-hands form an interaction with the III-IV linker that is disturbed by disease-causing mutations

**DOI:** 10.1038/s41598-018-22713-y

**Published:** 2018-03-14

**Authors:** Bernd R. Gardill, Ricardo E. Rivera-Acevedo, Ching-Chieh Tung, Mark Okon, Lawrence P. McIntosh, Filip Van Petegem

**Affiliations:** 1 Department of Biochemistry and Molecular Biology, The University of British Columbia, 2350 Health Sciences Mall, Vancouver, BC V6T 1Z3 Canada; 2 Department of Chemistry, The University of British Columbia, 2350 Health Sciences Mall, Vancouver, BC V6T 1Z3 Canada; 3 Present Address: Department of Anesthesiology, Pharmacology, and Therapeutics, The University of British Columbia, 2176 Health Sciences Mall, Vancouver, BC V6T 1Z3 Canada

## Abstract

Voltage-gated sodium channels (Na_V_) are responsible for the rapid depolarization of many excitable cells. They readily inactivate, a process where currents diminish after milliseconds of channel opening. They are also targets for a multitude of disease-causing mutations, many of which have been shown to affect inactivation. A cluster of disease mutations, linked to Long-QT and Brugada syndromes, is located in a C-terminal EF-hand like domain of Na_V_1.5, the predominant cardiac sodium channel isoform. Previous studies have suggested interactions with the III-IV linker, a cytosolic element directly involved in inactivation. Here we validate and map the interaction interface using isothermal titration calorimetry (ITC) and NMR spectroscopy. We investigated the impact of various disease mutations on the stability of the domain, and found that mutations that cause misfolding of the EF-hand domain result in hyperpolarizing shifts in the steady-state inactivation curve. Conversely, mutations in the III-IV linker that disrupt the interaction with the EF-hand domain also result in large hyperpolarization shifts, supporting the interaction between both elements in intact channels. Disrupting the interaction also causes large late currents, pointing to a dual role of the interaction in reducing the population of channels entering inactivation and in stabilizing the inactivated state.

## Introduction

Mammalian voltage-gated sodium channels (Na_V_s) are large plasma membrane proteins that selectively conduct Na^+^ ions down their electrochemical gradient^1^. In doing so, Na_v_s are responsible for the rapid depolarization of the plasma membrane in excitable cells.

**Figure 1:**
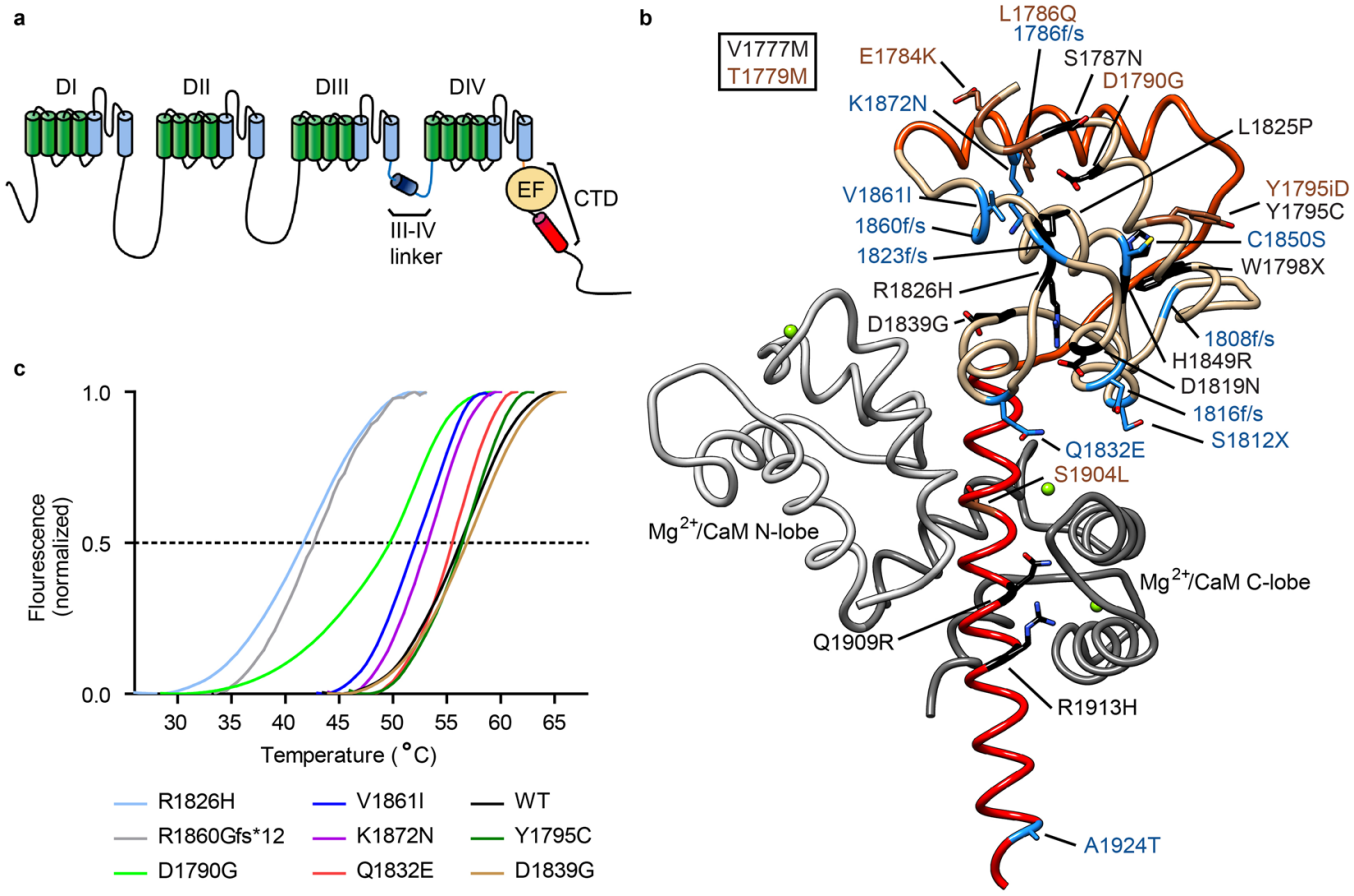
Disease mutations in the C-terminus of Na_v_1.5. (**a**) Schematic depiction of the structural entities of the channel. (**b**) Disease-associated mutations plotted on Na_v_1.5 C-terminal domain (red, light brown) interacting with Mg^2+^-loaded CaM (grey; PDB ID 4OVN). This region is immediately downstream of the last transmembrane segment and contains the EF-hand domain (beige), an additional helix (orange), and an IQ motif (red) that binds CaM. The disease-causing mutations are linked to LQT3 (black), BrS (blue), or both (brown). (**c**) Thermal denaturation curves for wild type and disease mutant EF-hand domain constructs using a Thermofluor assay. The displayed curves are an average of at least three measurements. The melting temperatures, standard errors, and number of repeats are shown in Table S1. R1860Gfs*12 is a frameshift mutation, which leads to a premature stop of translation after 11 amino acids of non-WT sequence.

Nine different pore-forming α-subunits exist (Na_V_1.1-1.9), which consist of four homologous repeats (I-IV) with 6 transmembrane helices each (S1-S6). Within each repeat, helices S1-S4 constitute a voltage-sensing domain (VSD), whereas helices S5 and S6 contribute to the pore (Fig. 1a). These repeats are linked by cytosolic loops with predicted intrinsic disorder. Near-atomic resolution cryo-electron microscopy (cryo-EM) structures have been solved for a putative Na_v_ channel from American cockroach^2^, and of Na_V_1.4 purified from electrical eel^3^. The mammalian α-subunit can associate with one or more of four different β-subunit isoforms^4^. These form single-transmembrane helices, with an extracellular immunoglobulin-like domain^3,5–7^ and a short cytosolic tail.

Several high-resolution structures have also been described for the C-terminal portion of mammalian Na_V_s^8–14^ (Fig. 1b). These show an EF-hand domain after the last transmembrane segment, followed by an IQ motif. The EF-hand domain forms a direct interaction site for fibroblast growth factor homologous factors^10,11^. Despite the presence of high Ca^2+^ concentrations in conditions used for multiple crystallography studies, no Ca^2+^ has been observed to bind to it. This is in contrast with previous experiments suggesting direct binding of Ca^2+^ to the EF-hand domain^15^. The IQ motif can bind Calmodulin (CaM) in its apo, as well as Mg^2+^ and Ca^2+^ loaded states.

Na_V_s inactivate fast, a process whereby ionic currents decrease dramatically within milliseconds of channel opening. Inactivation can also occur from the closed state, and the precise degree is voltage-dependent, increasing at more depolarized voltages. In the absence of high-resolution structures, the exact nature of the Na_V_ inactivated state remains to be described, but a large body of evidence points to the linker connecting repeats III and IV (III-IV linker) as a crucial element. Mutation of three consecutive hydrophobic residues – I,F,M – in this linker is sufficient to knock out fast inactivation, and an attractive model suggests it may bind to the transmembrane region, directly or indirectly occluding the pore^16,17^. The III-IV linker is therefore often referred to as an ‘inactivation gate’. In addition, several functional experiments have also hinted at a role for the C-terminal region in inactivation^18,19^.

Despite the crucial role of the III-IV linker in channel inactivation, its precise interactions within the Na_V_1.5 channel have remained undetermined by direct structural methods. In the absence of such structural insights, identifying these interactions is a challenging task, as they may be inherently weak, only occurring by virtue of a high local concentration of discontinuous channel segments. Here we utilize isothermal titration calorimetry (ITC) and Nuclear Magnetic Resonance (NMR) spectroscopy to dissect an interaction between the Na_V_1.5 III-IV linker and the EF-hand domain. Combined with functional assays, we propose a model whereby the interaction is required to maintain the proper voltage-dependence of inactivation.

## Results

### Disease mutations can destabilize the EF-hand domain

Na_V_1.5 is the target for multiple mutations linked to Long-QT type 3 (LQT3) and Brugada Syndromes^20–22^, several of which are in the EF-hand region (Fig. 1). Several are point mutations and we hypothesized that some of these could cause an intrinsic destabilization of the domain, affecting its inherent function. The EF-hand domain can be purified and is stable and monomeric on its own^9,13,23^. We produced wild-type and various mutant versions and measured their thermal stabilities via Thermofluor experiments (Fig. 1c, Supplementary Table S1). Wild-type EF-hand domain has a midpoint of unfolding temperature (T_m_) of 56 °C. Multiple point mutants have T_m_ values similar to the wild-type domain, but two of them, D1790G and R1826H, are destabilized by 6 °C and 14 °C, respectively. This can be explained by their location: D1790 is involved in a hydrogen bond with the amide hydrogen of S1787, which is lost by the D1790G substitution. The D1790G mutation has been linked to both LQT and Brugada syndromes^24,25^. R1826H has been linked to LQTS and sudden infant death syndrome^26,27^. R1826 forms a salt bridge with D1819 stabilizing the fold in all of the current structures of the C-terminal domain (CTD) determined by X-ray crystallography and NMR spectroscopy. R1860Gfs*12 introduces a frame shift, resulting in 11 residues of non-WT sequence and a preliminary stop. It results in a 14 °C decrease in thermal stability. This mutation omits large parts of the CTD and has been linked to sick sinus syndrome and atrial arrhythmias^28^.

The point mutants with the largest impact are L1825P and Y1795insD, because both resulted in misfolded EF-hand domain that was aggregated during expression and could not be purified in a monomeric form. L1825P has been linked to LQT and Brugada syndromes^29^ and affects a buried leucine residue, thus destabilizing the hydrophobic core. Y1795insD has been linked to LQT and Brugada syndromes^30,31^. It introduces an extra Asp residue immediately following Y1795. Since the latter is located in an alpha-helical region, all residues following this would thus be in a different register and experience different environments. The structural environment of both L1825 and Y1795 can thus explain the drastic effect on the folding stability.

### Mutations that cause misfolding of the EF-hand domain result in shifts of the steady-state inactivation curve

**Figure 2:**
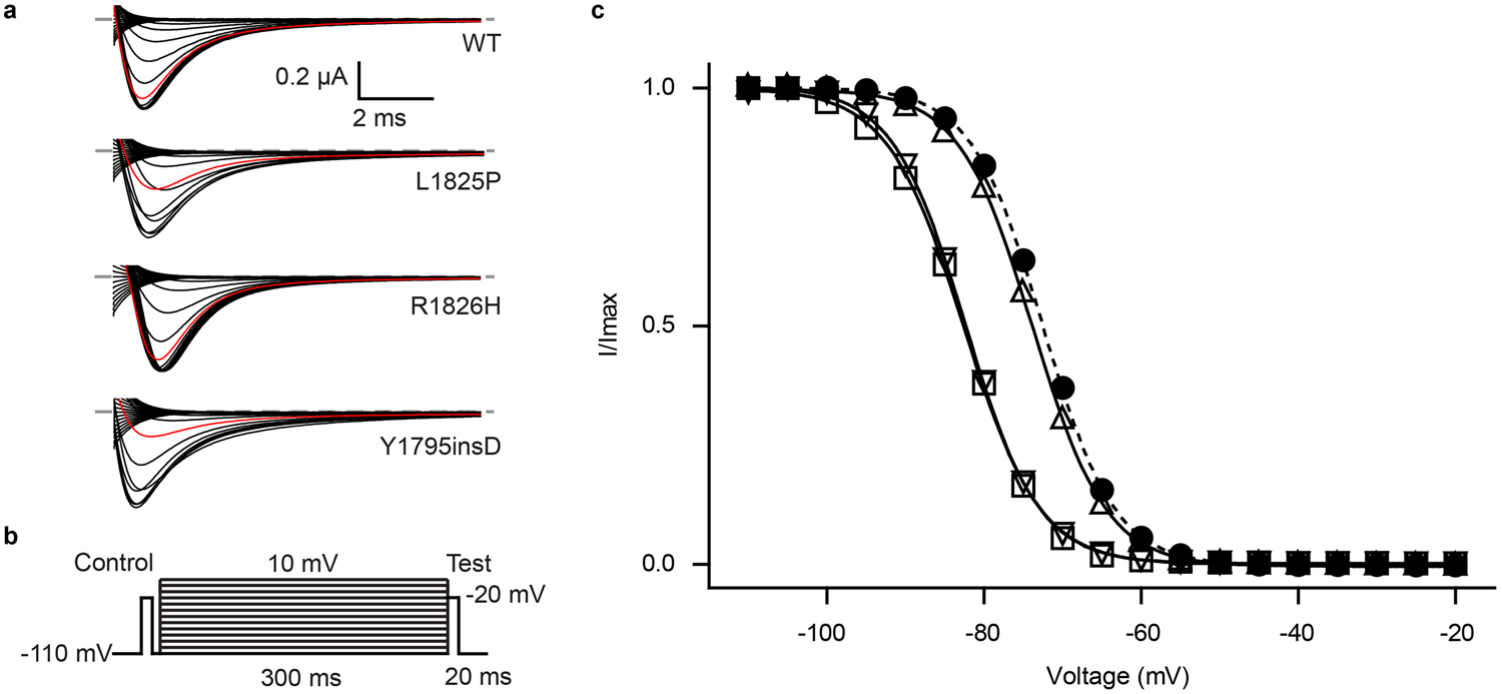
Inactivation-voltage relationship for wild-type and mutant Na_v_1.5 channels determined by two-electrode voltage clamp. (**a**) Representative raw traces of the test pulses for wild-type and disease mutants. The trace with −80 mV pre-pulse potential is shown in red. (**b**) Pulse protocol used to obtain steady-state inactivation curves. (**c**) Steady-state inactivation curve of wild-type (•),L1825P (□), R1826H (Δ) and Y1795insD (∇). The lines represent fits of the data with a Boltzmann equation. V_1/2_ values for inactivation are presented in Table S2.

Because of the drastic effect of both L1825P and Y1795insD, we decided to revisit their functional impact using two-electrode voltage clamp (TEVC). These were done in the presence of the Na_V_β1 subunit. We found no significant effect on the conductance-voltage (G-V) relationships. However, both mutants caused a general decrease in current amplitude (Supplementary Fig. S1), an effect not surprising given their general impact on folding. In addition, both mutants also caused a robust ~9 mV hyperpolarizing shift in the steady-state inactivation curve (V_1/2,inact._ for WT: −73.13 ± 0.07 mV, n = 9; L1825P: −82.59 ± 0.42 mV, n = 7; Y1795insD: −82.25 ± 0.98 mV, n = 8) (Fig. 2 and Supplementary Table S2). This suggests a role for the EF-hand domain in reducing the extent of inactivation. Although we cannot exclude that the domain retains structure in full-length channels, our observation that the mutations interfere with folding of the isolated domain suggest a relatively large perturbation. This would then interfere with the ability of the domain to reduce the extent of inactivation, resulting in more inactivation at hyperpolarized potentials.

Since R1826H results in a large shift in temperature stability, we also measured its steady-state inactivation curve. No significant shift was found relative to wild type (V_1/2,inact._ = −73.67 ± 0.15 mV, n = 13). However, since this mutant only starts to unfold at temperatures above 30 °C, it is conceivable that the steady-state inactivation behaviour is different from wild-type at physiologically relevant temperatures.

### Interaction between the EF-hand domain and the III-IV linker

**Figure 3:**
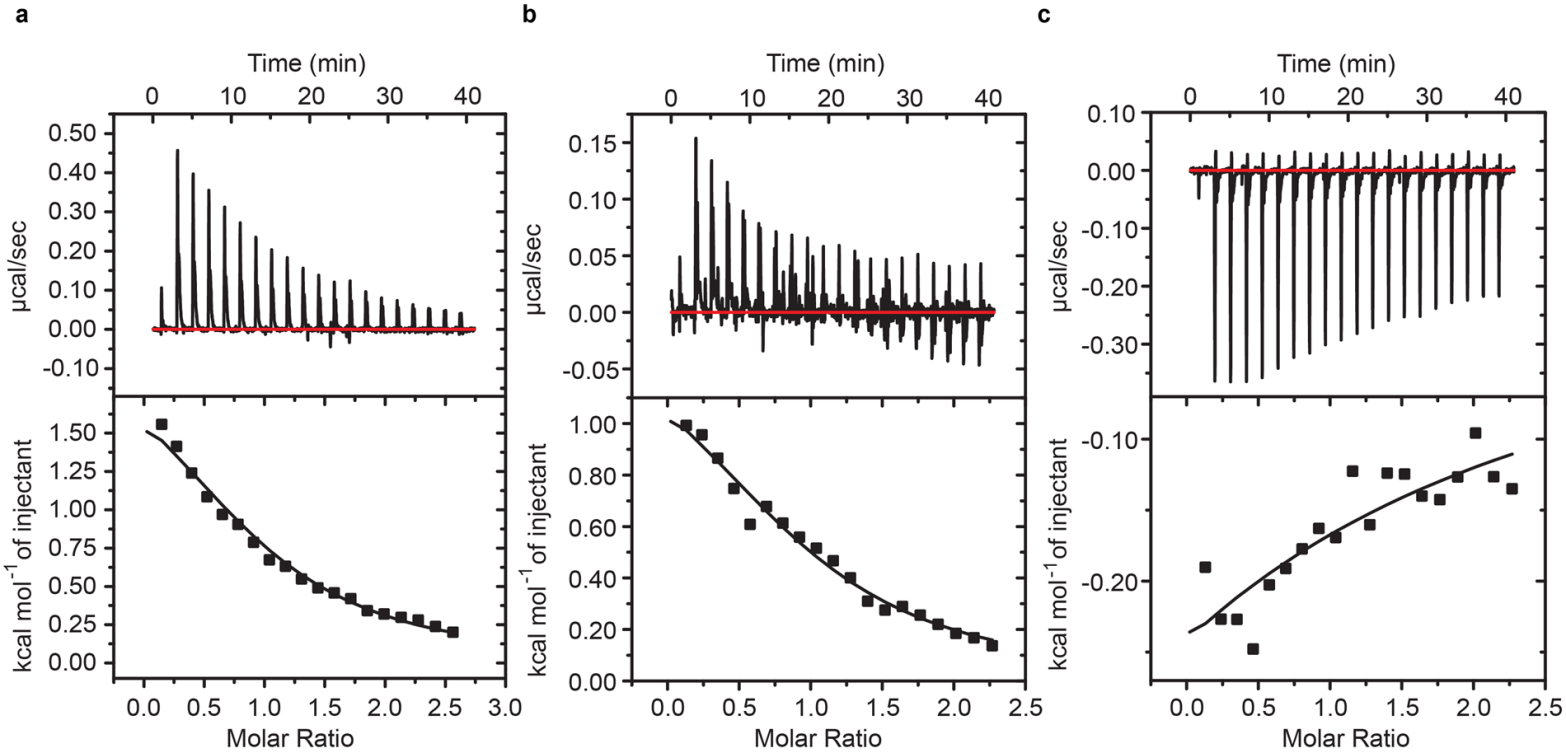
Isothermal titration calorimetry data of the EF-hand domain interaction with the III-IV linker. (**a**) EF-hand domain (160 μM) was titrated with aliquots of 2 mM III-IV linker. Average of three repeats yielded a *K*_d_ of 87 μM ± 6 μM. (**b**) III-IV linker (175 μM) was titrated with 2 mM EF-hand domain in buffer containing 4 mM CaCl_2_. One set of sites model fit in Origin yielded a *K*_d_ of 87 μM ± 12 μM. (**c**) III-IV linker (175 μM) premixed with 285 μM of CaM titrated with EF-hand domain in buffer containing 4 mM CaCl_2_. These show that the interaction now is exothermic instead of endothermic. The integrated heats are too small to allow a reliable fit of the data.

Because the III-IV linker has previously been shown to play a key role in inactivation, a logical hypothesis is that it may interact with the EF-hand domain. Indeed, previous pull-down experiments have suggested such an interaction^19^, but no quantitative analysis or direct mapping of the interface have thus far been performed. We therefore purified polypeptides corresponding to both the III-IV linker and the EF-hand domain independently. Both constructs appeared well-behaved and monomeric in size exclusion chromatograms, a pre-requisite for quantitative binding experiments. We determined their affinities via isothermal titration calorimetry (ITC), yielding a *K*_d_ of 87 μM ± 6 μM (Fig. 3a). The interaction has a positive enthalpic change (ΔH = 2200 ± 100 cal.mol^−1^) and is thus entirely driven by entropy (ΔS = 26 ± 1 cal.mol^−1^.deg^−1^). The reported values are an average of three repeats, stating the standard deviation. Although the affinity seems modest, the local concentration of both segments within full-length channels could be very high. Indeed, recent cryo-EM structures of Ca_V_1.1 and a cockroach Na_V_ show immediate proximity between the EF-hand and III-IV linker^2,32^.

**Figure 4:**
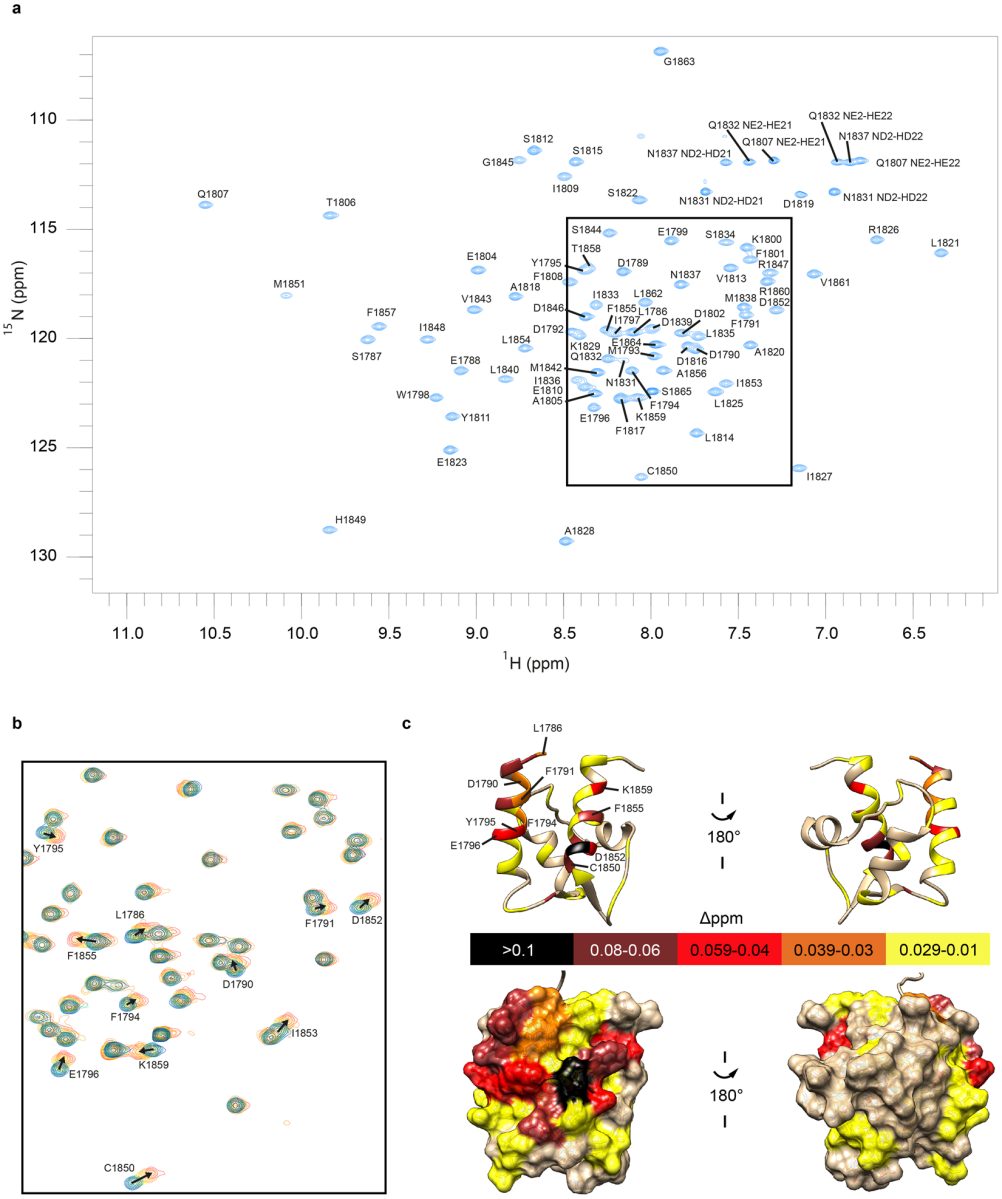
NMR spectroscopic characterization of the EF-hand domain interaction with III-IV linker peptide. (**a**) Assigned ^15^N^−^HSQC spectrum of the ^15^N/^13^C-labelled EF-hand domain. (**b**) Superimposed ^15^N^-^HSQC spectra (boxed region in A) showing chemical shift perturbations of the ^15^N-isotopically labelled EF-hand domain before and after addition of the III-IV linker peptide. Spectra are coloured according to the molar ratio of EF-hand domain: III-IV linker, 1:0 blue, 1:0.25 cyan, 1:0.5 green, 1:1 yellow, 1:2 orange, 1:4 red. Annotated peaks show weighted amide ^1^H^N^-^15^N shift changes of more than 0.03 ppm at the final titration point. (**c**) Weighted chemical shift perturbations mapped on the EF-hand domain structure 2KBI. The ribbon representation is annotated with residues corresponding to peaks found to shift. The bottom panel shows a surface representation.

Next we utilized NMR spectroscopy to map the interaction site for the III-IV linker on the EF-hand domain. Because the exact construct differs from that used for a previously published NMR-derived structure^13^, we first produced ^15^N/^13^C-labeled EF-hand domain to assign the signals from its main chain ^1^H, ^13^C and ^15^N nuclei (Fig. 4a). The well-dispersed spectra confirm that the current EF-hand domain adopts a stable fold when separated from its native context in Na_V_1.5. To determine residues involved in binding interactions, we then titrated a sample of ^15^N-labeled EF-hand domain with unlabelled III-IV linker peptide in five steps to a final 4-fold molar excess.

Figure 4b shows a close-up of the spectral region where many peaks exhibited chemical shift perturbations. In the presence of excess III-IV linker, relatively small amide chemical perturbations are observed for the EF-hand domain. This suggests that the amide backbone of the EF-hand domain undergoes little structural perturbations upon peptide binding. The progressive changes in amide chemical shifts with increasing concentrations of peptide indicates that binding occurs in the fast exchange regime^33^. We mapped the perturbations on the previously reported structure of the EF-hand domain (Fig. 4c). Amides that show spectral perturbations cluster on one surface of the EF-hand domain. This serves to define the III-IV linker binding interface. Interestingly, many of the perturbed residues are negatively charged, suggesting that a major part of the interaction may be driven by electrostatic interactions. Because of the higher ionic strength buffer used for the NMR experiments, we expect a weaker affinity if the binding is indeed dependent on ionic interactions. Consistent with this, the EF-hand domain and III-IV linker peptide show qualitatively weaker binding with a *K*_d_ of about 900 μM. Several residues with perturbed chemical shifts, including L1786, S1787, D1790, Y1795, and C1850, have been previously associated with disease-causing mutations^24,25,30,31,34–37^. Altered interactions between the EF-hand domain and III-IV linker may thus be responsible for part or all of the disease phenotype.

### Identifying the binding site on the III-IV linker

**Figure 5:**
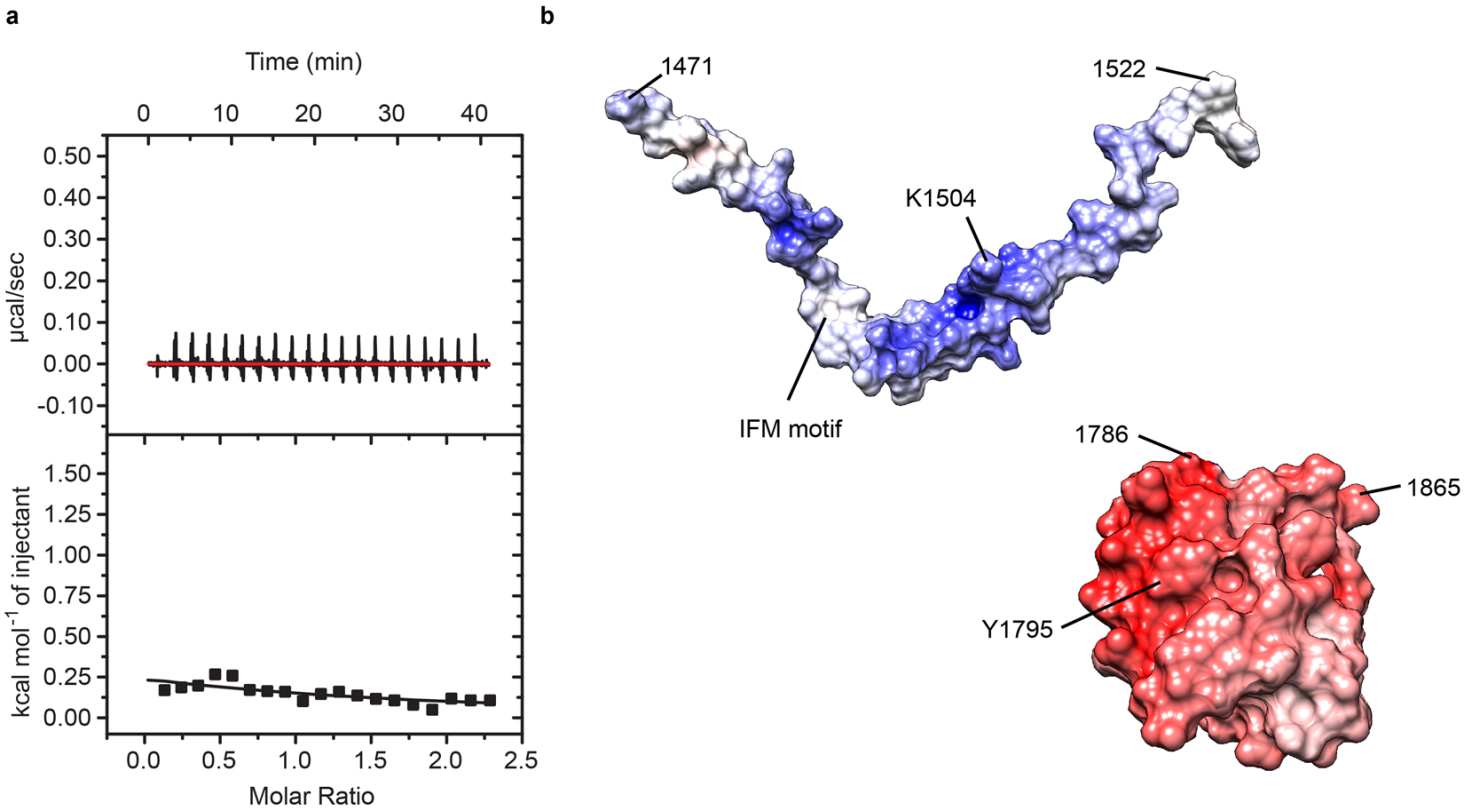
Isothermal titration calorimetry data demonstrating that the EF-hand domain does not interact with III-IV linker mutant K1504E/K1505E. (**a**) Purified EF-hand domain sample at 175 µM was titrated with 2 mM K1504E/K1505E peptide. Compared with Fig. 3, the mutation clearly affects binding. The binding isotherm is too shallow to allow a reliable fit. (**b**) Theoretical electrostatic surface potential of the interaction partners plotted on models of III-IV linker (PDB ID 1BYY, extended for missing Na_v_1.5 sequence via Modeller^61^) and EF-hand domain (PDB ID 2KBI, shown are only residues included in our constructs). Potentials calculated with the APBS tool in UCSF Chimera, red indicates negative, blue positive charge.

The III-IV linker has previously been shown to contain an α-helical segment but otherwise is intrinsically disordered in isolation^38,39^. Unfortunately, high quality ^15^N-HSQC spectra with well-dispersed peaks could not be obtained for a labelled III-IV linker polypeptide. The electrostatic surface potential of the EF-hand domain is predominantly negative (Fig. 5b), with multiple negatively charged residues undergoing chemical shifts in the NMR titration. The III-IV linker, on the other hand, has an overall positive electrostatic potential, suggesting these may be involved in binding. Charge reversals of these III-IV linker residues were then investigated via ITC to see if they are involved in the interaction (Figs 5 and S2). The greatest impact was found by mutating the lysine pair K1504/K1505 to glutamates, as we could no longer detect an interaction with the EF hand under identical conditions used for the wild-type.

The III-IV linker was previously shown to contain binding sites for Ca^2+^/CaM^23,39–41^. We therefore tested whether the binding sites for CaM and the EF-hand domain overlap via a competition experiment. First we tested a titration of III-IV linker with EF-hand domain in the presence of Ca^2+^ (Fig. 3b). The obtained *K*_d_ of about 90 μM closely resembles the value obtained for the opposite titration in the absence of Ca^2+^ (Fig. 3a). We then pre-mixed Ca^2+^/CaM with the III-IV linker before titration with the EF-hand domain. This interaction now proceeds with a negative enthalpy change, instead of a positive change (Fig. 3c). Although the ITC still suggests binding of the EF-hand to the III-IV linker in the presence of CaM, the interaction is too weak to obtain a reliable fit. This demonstrates that there is a reduced affinity in the presence of Ca^2+^/CaM, indicating a degree of steric hindrance and hence competition. Of note, CaM only binds the III-IV linker in the presence of Ca^2+^, so no competition would be expected between apoCaM and the EF-hand domain.

**Figure 6:**
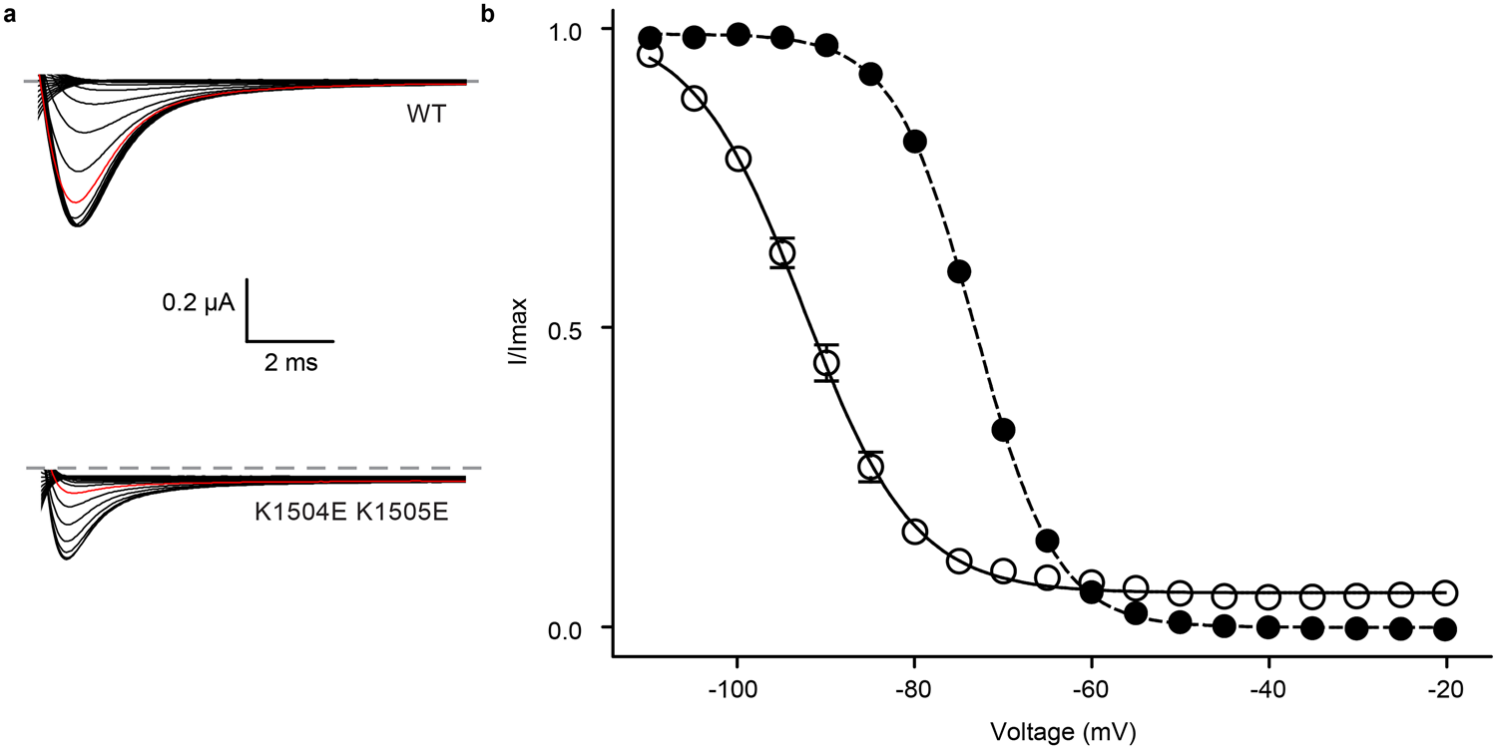
Two-electrode voltage clamp measurement of steady-state inactivation shows differences in voltage-dependent inactivation for designed inactivation gate mutant. (**a**) Representative raw traces of the test pulse for wild-type and K1504E K1505E Na_V_1.5. The −80 mV pre-pulse potential trace is shown in red. (**b**) Steady state inactivation data of wild-type (•) and mutant channels (ο) fitted with a Boltzmann function. The holding potential was −110 mV. The mutant shows a pronounced difference in inactivation and late sodium current.

We next investigated the functional impact of the K1504E/K1505E double mutant using TEVC experiments in presence of Na_v_β1. A small depolarizing shift is visible for the I-V curve (Fig. S3), but the largest effect was found for the steady-state inactivation curve, with a 19.5 mV hyperpolarizing shift (Fig. 6). This mirrors the effect of the mutations that cause misfolding of the EF-hand domain, although with a higher magnitude. The most straightforward explanation is that the interaction between the EF-hand domain and the III-IV linker reduces the relative population of the III-IV linker conformation that enables inactivation. Breaking this interaction by mutations on either side thus produces more inactivation.

However, another effect is that the inactivation at more depolarized potentials does not saturate to zero. As it is clear from both the steady-state inactivation curve and on individual traces, the currents remain at a level of ~9% (at −30 mV) of the peak current (Supplementary Fig. S3). This so-called late sodium current has been implicated in cardiac arrhythmia, underlying Long-QT syndrome type 3^37,42^. It thus appears that the interaction between the III-IV linker and EF-hand domain has a dual role in preventing inactivation at hyperpolarized potentials, and in producing a stable inactivated state^19^.

## Discussion

Na_V_s have the intriguing property to inactivate very fast, whereby macroscopic currents decay to almost zero within milliseconds of opening. Na_V_ inactivation is thought to require activation of the voltage-sensing domain (VSD) of Repeat IV^1,43–45^. This also allows the channel to become inactivated prior to opening. Indeed, a steady-state inactivation curve shows the availability of Na_V_s as a function of different holding potentials, showing a clear voltage-dependence and a large extent of inactivation at voltages where the channels are still predominantly closed. Tailoring the exact voltage-dependence of this curve can thus dictate the amount of available sodium channels.

The III-IV linker has received great attention as a primary segment involved in channel inactivation. Mutations in the hydrophobic ‘IFM’ motif, located within the N-terminal part of the linker, can obliterate fast inactivation. This has led to the hypothesis that this motif can form a hydrophobic latch, possibly blocking the pore^17^. However, a recent cryo-EM derived structure of Na_V_1.4 from electric eel (EeNa_V_1.4) showed the IFM motif to interact with a pocket lined by the outer S4-S5 and inner S6 segments in repeats III and IV, suggesting an allosteric mechanism rather than a direct block^3^.

Here we show that the EF-hand domain can associate with the III-IV linker, supporting previous pull-down experiments^19^. In solution, this interaction proceeds with a *K*_d_ of ~90 μM, as shown by ITC experiments. Several pieces of evidence suggest that this interaction also occurs within full-length Na_V_1.5. First, the functional effects, obtained by disrupting the interaction from either side, mirror one another. On the EF-hand side, either Y1795insD or L1825P, both of which abolish folding, result in hyperpolarizing shifts in steady state inactivation and produce late sodium currents^29–31,46^. On the III-IV linker side, our results show that mutation of K1504/K1505 to a pair of glutamates abolishes the interaction with the EF-hand and causes a hyperpolarizing shift in steady-state inactivation and very large late sodium currents. The extent of the shift and the late current appears much bigger for the K1504E/K1505E double mutant, suggesting a larger impact on the interaction. This could be explained by the amount of channels in which the EF-hand domain is misfolded for the Y1795insD and L1825P mutants: although we cannot produce pure folded EF-hand domain for these two disease mutants in *E*. *coli*, it is possible that a fraction of the EF-hands in full-length channels is still in a folded state. The steady-state inactivation curve shows the aggregate behaviour of all functional Na_V_1.5 channels at the plasma membrane, and could thus be an average of channels where the EF-hand is either folded or misfolded. On the other hand, the K1504E/K1505E double mutation would abolish the interaction for all channels, thus showing a more pronounced shift of the steady-state inactivation curve and larger late currents.

**Figure 7:**
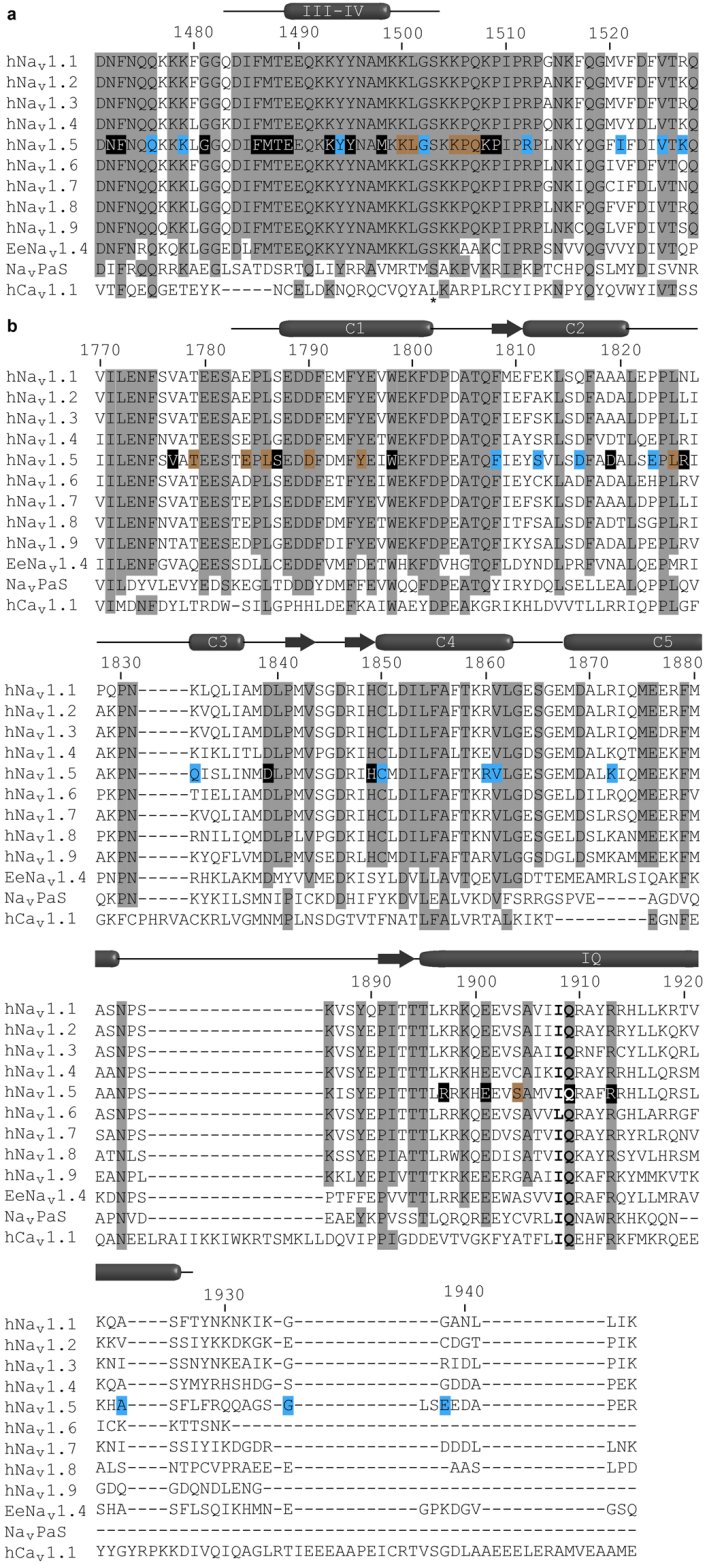
Sequence alignment of mammalian voltage-gated sodium channel isoforms with Na_v_PaS, EeNa_v_1.4 and human Ca_V_1.1. (**a**) III-IV linker region and (**b**) C-terminal domain region aligned with Clustal Omega. Fully conserved residues are shaded. Known disease associated residues in Na_v_1.5 are marked by boxes coloured according to their disease phenotype LQT3 (black), BrS (blue), or both (brown). The phosphorylation site in the III-IV linker is marked by an asterix^51^. Secondary structure has been annotated according to the DSSP calculations of Na_v_1.2 (PDB ID 1BYY) and Na_v_1.5 (PDB ID 4OVN) structures in the protein structure database.

A second piece of support comes from recent cryo-EM derived structures of a skeletal muscle voltage-gated calcium channel (Ca_V_1.1)^32^ and a cockroach sodium channel (Na_V_PaS)^2^. In both cases, the EF-hand domain was found to interact directly with the III-IV linker, despite significant divergences in sequence between both channels. The III-IV linker in Ca_V_1.1 is significantly shorter, so it is hard to use this as a template to map mutations. The Na_V_PaS III-IV linker, however, has the same length as in Na_V_1.5, displaying 33% sequence identity, whereas the EF-hand domain shows 37% identity. Importantly, mapping of the residues equivalent to the pair K1504/K1505 shows that they are in direct contact with the EF-hand domain, thus explaining how mutating this site abolishes the interaction. Together with the functional data, we thus propose that an interaction between the EF-hand domain and III-IV linker is conserved among different channels, despite significant sequence divergence. Given the high sequence conservation for the mammalian Na_V_s in this area (Fig. 7), it is likely that this interaction occurs for all nine isoforms, but this remains to be verified.

**Figure 8:**
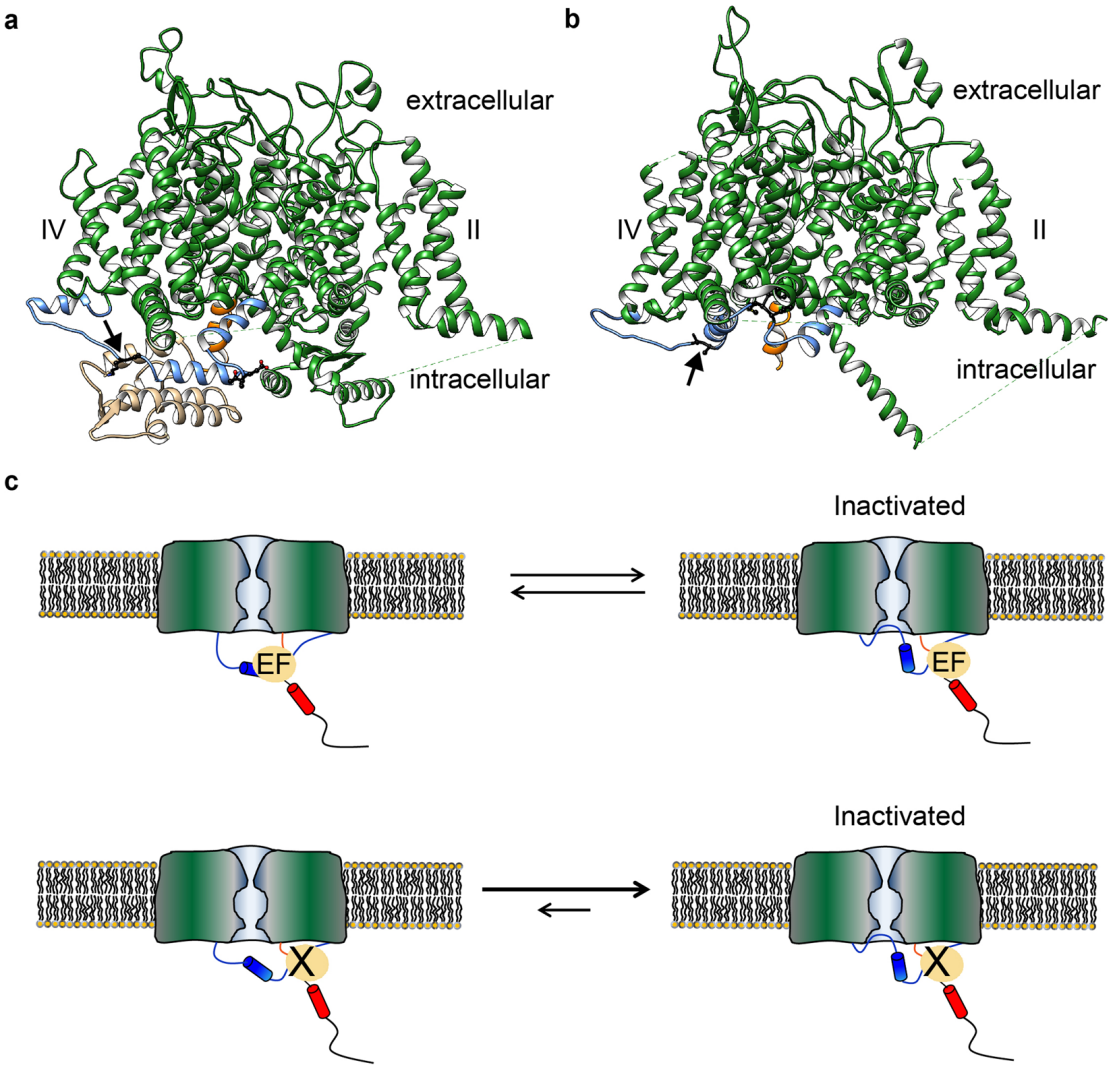
Model for the functional importance of the EF-hand domain: III-IV linker interaction. (**a**) Structure of Na_V_PaS (PDB ID 5X0M) showing the III-IV linker (blue) in close contact with the EF-hand domain (beige). (**b**) In the structure of EeNa_v_1.4 (PDB ID 5XSY) the EF-hand like domain is not resolved and the III-IV linker (blue) adopts a conformation where the IFM equivalent is interacting with a hydrophobic pocket, possibly representing an inactivated state. The EF-hand domain is not present in the structural model indicating possible mobility. Subunits other than the pore forming α-subunit have been omitted for clarity. In both structures the equivalents of the IFM motif are depicted as black sticks. The arrows indicate the position of the engineered mutation site (K1504E/K1505E in Na_V_1.5). Voltage sensors of domain II and IV are labelled; domain III is oriented towards the viewer. For ease of orientation the end of helix 6 transmembrane domain IV is coloured orange. (**c**) The EF hand domain interacts with the III-IV linker, thus imposing an energetic barrier for channel inactivation at a given membrane potential. Abolishing the interaction (bottom), either through disease mutations in the EF-hand domain at the interface, or by targeted mutations in the III-IV linker, results in more channel inactivation. Paradoxically, this also allows the channel to go into a different state resulting in late sodium currents.

How would the EF-hand domain modulate inactivation? Currently no structures are available for the same Na_V_ in both inactivated and non-inactivated states. However, a direct comparison of the Na_V_PaS and EeNa_V_1.4 cryo-EM derived structures suggests a possible mechanism (Fig. 8). Both were solved in the absence of any electrical field, which would promote an inactivated state^3^. The Na_V_PaS misses the conventional IFM motif and may thus represent a channel that does not inactivate. Making these assumptions, it thus seems that the III-IV linker binds the EF-hand in channels that are not inactivated (Na_V_PaS), but instead interacts with the transmembrane region in inactivated channels (EeNa_V_1.4). In the latter case, the EF-hand domain shows only weak cryo-EM density, suggesting it has increased mobility and a more dynamic interaction with the III-IV linker^3^. Breaking the interaction with the EF hand via targeted mutations or disease variants would thus allow inactivation to proceed more readily, resulting in a hyperpolarizing shift in steady-state inactivation (Fig. 8).

An important but puzzling finding is that the interaction appears to modulate Na_V_1.5 inactivation in two ways. The first one is to modulate the availability of sodium channels, by causing a depolarizing shift of the steady-state inactivation curve. A second role for the interaction may be to stabilize the inactivated state, since the K1504E/K1505E double mutant produces very pronounced late currents. Indeed, it has previously been suggested that an interaction between the III-IV loop and the C-terminal region of Na_V_1.5 may be necessary to minimize channel reopening^19^. Interestingly, the mutations that cause misfolding of the Na_V_1.5 EF-hand domain have previously also been shown to result in increased late currents: L1825P results in ~2.5–5% of late current compared to 0.2–0.3% for wild type depending on the chosen time point^29,46^. For Y1795insD, one report suggests a late current of ~1.4%, but this went undetected in a report by a different group^30,31^. Two other disease mutations at this residue, Y1795H and Y1795C, both show late sodium currents^36^. The archetypical disease mutation in the III-IV linker ΔKPQ which results in an in-frame deletion of K1505, P1506 and Q1507^20^, shows a −5.8 mV shift in V_1/2_ steady-state inactivation and a late sodium current smaller than 5% of the peak current^47^. The precise extent of change in late current may be related to the impact of the variants on the interaction with the EF-hand domain. In our case, we engineered a K1504E/K1505E double mutant for which we could no longer detect an interaction with the EF-hand domain, leading to a very large late current of 9%.

Our thermal denaturation data show that some disease mutations in the EF-hand domain cause a large shift in thermal stability, with the most pronounced effect for R1826H. In contrast to L1825P and Y1795insD, we did not observe a depolarizing shift in steady-state inactivation. However, the T_m_ for this mutant is ~42 °C (Table S1), with visible rise in the denaturation curve only occurring above 30 °C. The inactivation experiments were performed at room temperature, so no effects on the inactivation are expected. However, this does suggest the possibility to see differences compared to wild-type at physiologically relevant temperatures.

Using NMR spectroscopy, we mapped the interface of the III-IV linker on the Na_V_1.5 EF-hand domain. Residues showing chemical shift perturbations upon linker binding include a number of sites for mutations associated with Brugada and Long-QT syndromes, namely L1786Q, S1787N, D1790G, Y1795, C1850^24,25,30,31,34–37^. Table S3 outlines the disease phenotypes and reported functional impacts. Importantly, several of these cause shifts in the steady-state inactivation curve, increased late currents, or both. This underlines the susceptibility of the interaction site for disease mutations which might alter the strength of the interaction between EF-hand domain and III-IV linker and therefore the fine balance of inactivation. These observations are consistent with our hypothesis that the EF-hand:III-IV linker interaction regulates both steady-state inactivation and increased late current.

The binding surface we describe here for on the EF-hand domain may also be targeted by another channel segment. Using both NMR spectroscopy and ITC experiments, it has previously been shown that the EF-hand domain can interact with the IQ motif using the same side of its surface as the one we describe^13^. The IQ motif also binds CaM in both the absence and presence of Ca^2+^, and to date all crystal structures of C-terminal Na_V_ fragments show no intramolecular interaction between IQ motif and EF-hand domain. Such an interaction is therefore only likely to occur for the fraction of channels not bound to CaM, resulting in a possible competition between the III-IV linker and the IQ motif for binding to the EF-hand domain.

The interaction between III-IV linker and EF-hand domain may be regulated, since both have also been found to interact with auxiliary proteins. The EF-hand domain is able to interact with fibroblast growth factor homologous factors (FHF)^10,11^ and there are data suggesting a direct interaction of the α-subunit and β1 close to D1790 and E1784^24,48^. CaM can bind to the IQ motif immediately C-terminal to the EF-hand domain, under Ca^2+^-loaded or Ca^2+^ free conditions and crystal structures show additional interactions between CaM and the EF-hand domain^8,49,50^. Post-translation modifications may also affect the interaction. For example, S1503 has been identified as a target for phosphorylation by CaMKII^51^. It is adjacent to our engineered K1504E/K1505E pair that knocks out the interaction. Interestingly, phosphorylation of this residue also leads to a hyperpolarizing shift in the steady-state inactivation^51^, suggesting that it may also act by affecting the interaction between the III-IV linker and the EF-hand domain.

The ability of the EF-hand domain to interact with the III-IV linker is reminiscent of a previous report whereby the CaM C-lobe can interact with the same linker in a Ca^2+^-dependent manner^23,39–41,52^. This suggests that the EF-hand:III-IV linker interaction may be disrupted or altered under high Ca^2+^ conditions. The precise binding site for Ca^2+^/CaM is upstream of the K1504/K1505 pair, suggesting that EF-hand domain and Ca^2+^/ C-lobe may bind simultaneously. However, our competition experiments show altered affinities, suggesting some degree of steric hindrance. Whether Ca^2+^/CaM can truly displace the EF-hand domain from the III-IV linker remains to be found, as it will also depend on the local concentration of either one in full-length channels. It also is possible that the binding of Ca^2+^/CaM is functionally redundant to the binding of the EF-hand, since binding of either results in depolarizing shifts of the steady-state inactivation curve. Further biochemical and structural investigation will undoubtedly shed more light on this.

## Methods

### Cloning and mutagenesis

Cloning of the human Na_V_1.5 III-IV linker construct for biochemistry was described previously^52^. The human Na_V_1.5 EF-hand domain construct for NMR spectroscopy (residues 1786–1865), the EF-hand domain for Thermofluor experiments (residues 1786–1898), and CaM were cloned from human cDNA into a modified pET-28 vector^53^. Disease mutations in these constructs were introduced following the QuikChange protocol (Stratagene).

The constructs used for electrophysiology experiments were human Na_v_1.5 α-subunit (dbSNP:rs763891399)^54^ and rat β1-subunit (NM_001271045.1) in pBSTA vectors. Mutations in full-length constructs were introduced utilizing either QuikChange (Stratagene) or Q5® Site-Directed Mutagenesis Kit (New England BioLabs).

### Protein expression and purification

Na_v_1.5 constructs were expressed as His-MBP-tagged fusions with a TEV protease cleavage site from a modified pET-28 vector^53^ in *E*. *coli* Rosetta cells. EF-hand constructs bearing disease mutants were produced in 2xYT medium and those for NMR spectroscopic studies were expressed in ZYM-5052 auto-inducing medium (unlabelled), N-5052 auto-inducing^55^ (^15^N-labeled), or a modified N-5052 supplemented with 1 g/L thiamine HCl and 3 g/L ^13^C_6_-glucose. For non-auto-inducing media, expression was induced at OD_600_ of 0.5 by addition of 0.4 mM IPTG. Media containing 100 μg/L kanamycin and 34 μg/L chloramphenicol was inoculated with densely grown overnight culture and grown at 37 °C until it reached OD_600_ of 0.3, the temperature was then lowered to 30 °C and cultures grown to high density.

Cell pellets were resuspended in lysis buffer containing 10 mM HEPES pH 7.4, 250 mM KCl, 20 mM Imidazole. For efficient lysis and inhibition of proteases the suspension was supplemented with DNase, Lysozyme and PMSF. The cells were lysed by ultrasonication on ice, followed by centrifugation at 35,000 g for 30 min. The supernatant was applied to HisTrap FF columns (GE Healthcare), and the fusion protein was eluted with 10 mM HEPES pH 7.4, 250 mM KCl, 500 mM imidazole pH 7.4. The elution peak was loaded onto an amylose column (New England Biolabs) and washed with either 10 mM HEPES pH 7.4, 150 mM KCl for III-IV linker constructs, or 20 mM Tris-Cl pH 7.6 plus 10 mM NaCl for EF-hand constructs. TCEP was added to a final concentration of 2 mM, and the sample was incubated overnight at 4 °C after addition of TEV protease. The inactivation gate samples were then loaded on a Talon column (Clontech) and the flow-through containing the peptide was collected. A final step consisted of a Superdex 75 (GE Healthcare) column in 10 mM HEPES pH 7.4, 150 mM KCl. In the case of EF-hand purifications, as a second step, the samples were loaded onto a Q Sepharose HP column (GE Healthcare) and after washout of unbound sample an optimized gradient from 20 mM Tris-Base pH 7.6, 10 mM NaCl to 20 mM Tris-Base pH 7.6, 1.5 M NaCl was run. The peak containing the target protein was then concentrated and loaded on a Superdex 75 column (GE Healthcare) in 10 mM HEPES pH 7.4, 150 mM KCl for a polishing step.

Purification of CaM followed the same general procedures, while using the unique properties of CaM. After the initial HisTrap FF column (GE Healthcare), the protein was eluted and TEV protease was added for cleavage at 4 °C overnight in the presence of 2 mM TCEP. The next morning CaCl_2_ was added as a powder to a final concentration of 100 mM. The sample was then loaded on a Phenyl Sepharose column (GE Healthcare), washed with 10 mM HEPES pH 7.4, 150 mM KCl, 10 mM CaCl_2_ and eluted with 10 mM HEPES pH 7.4, 150 mM KCl, 10 mM EDTA. It was loaded on a Superdex 75 column (GE Healthcare) in 10 mM HEPES pH 7.4, 150 mM KCl and passed through a HisTrap FF column for removal of trace MBP.

Pure samples were concentrated in a 3 kDa MWCO Amicon centrifugal filter (Milipore). The expected masses of the purified samples were confirmed by mass spectrometry. Concentrations were determined by absorption at 280 nm.

### Thermofluor assay

Thermal denaturation curves of Na_v_ 1.5 residue 1786–1898 WT and mutants (D1790G, Y1795C, R1826H, Q1832E, D1839G, V1861I, or K1872N, R1860Gfs*12) were obtained by means of Thermofluor experiments^56^. Protein samples (50 μL) at 0.2 mg/mL were mixed with 1 × SYPRO Orange (Invitrogen) according to manufacturer’s instructions. Data was recorded in a DNA Engine Opticon 2 real-time PCR machine (Biorad), using the SYBR green filter option. A temperature gradient from 20 °C to 95 °C in 0.5 °C steps was measured, where each temperature was maintained for 15 s. The midpoint temperature of protein unfolding was determined by a fit using the Boltzmann function on the curves in GraphPad Prism (GraphPad Software).

### Isothermal titration calorimetry

All data were collected on an ITC200 instrument (GE Healthcare) at 5 °C. Purified protein samples were dialyzed against 10 mM HEPES pH 7.4, 150 mM KCl, 1 mM EDTA using a 1 kDa molecular weight cut-off membrane (Spectrumlabs). Typically, the EF-hand constructs were used at 175 μM concentration in the cell and they were titrated with 1.9–2.2 mM inactivation-gate peptide. For testing of competition with CaM binding, samples were dialyzed against 10 mM HEPES pH 7.4, 150 mM KCl, 4 mM CaCl_2_. A mixture of 175 μM inactivation-gate peptide and 285 µM CaM was titrated with 2 mM EF-hand construct. The data were analysed using the Origin Plugin (OriginLabs) provided with the instrument (GE Healthcare) for subtraction of reference and fitting of a standard one-binding site model.

### Nuclear magnetic resonance spectroscopy

Data were collected on a cryoprobe-equipped Bruker Advance III 500 MHz spectrometer at 25 °C. Both the ^15^N-labelled EF-hand domain and unlabelled III-IV linker peptide were exchanged to 100 mM Na-phosphate pH 6.5, 200 mM NaCl, 5 mM β-mercaptoethanol and 0.01% sodium azide, followed by the addition 5% (v/v) D_2_O lock solvent. Since the EF-hand domain construct differs from that previously studied by NMR spectroscopy^13^, standard ^1^H-^13^C-^15^N scalar correlation experiments^57^ were recorded with a ^13^C/^15^N-labelled sample (800 μM) to assign signals from main chain nuclei. The interactions of III-IV linker with EF-hand domain were monitored with ^15^N-HSQC spectra. Aliquots of unlabelled peptide (2 mM stock) were titrated into a sample of 200 μM ^15^N-labelled EF-hand domain at final molar ratios of 0.25:1, 0.5:1, 1:1, 2:1, and 4:1. The data were processed with NMRPipe^58^ and analysed either with Sparky 3.114 or CcpNmr 2.4.2^59^. The weighted amide ^1^H^N^-^15^N chemical shift perturbation were determined with the CcpNmr Follow Shift Changes tool using the standard settings, (Δδ = [(Δδ_1H,ppm_)^2^ + (0.15Δδ_15N,ppm_)^2]1/2^).

### Electrophysiology

All animal work has been conducted in accordance with Canadian council on animal care guidelines, through the University of British Columbia Animal Care Committee approved protocols (application number A13-0212). One adult female frog of *Xenopus laevis* was terminally anesthetised and lobes containing oocytes were surgically extracted. Oocytes were defolliculated by incubation with collagenase type Ia (Sigma-Aldrich) and washed in a modified Ringer’s solution (OR3) containing 50% (v/v) L15-Medium, 1 mM L-glutamine, 250 μg/mL gentamycin (Gibco) and 15 mM HEPES, adjusted to pH 7.6 with NaOH. Oocytes were then sorted for stage V-VI and kept in OR3. mRNA was prepared from linearized DNA using the mMessage mMachine® T7 Transcription Kit (ThermoFisher). A mixture of 100 ng/μL α-subunit and 75 ng/μL β1-subunit was used for injection of 3.2–8 ng RNA. Oocytes were incubated in OR3 at 18 °C for 24–72 h before measurements were performed in Ringer’s solution containing 5 mM HEPES pH 7.4, 116 mM NaCl, 2 mM KCl, 1.8 mM CaCl_2_, and 2 mM MgCl_2_. The electrodes were pulled from borosilicate glass capillaries using a Model P-97 Micropipette Puller (Sutter Instruments) and filled with 3 M KCl solution.

Data collection was conducted with an Axoclamp 900 A amplifier and a Digidata 1440 A Digitizer (Molecular Devices). To measure inactivation-voltage relationships the oocytes were held at −110 mV, and then given a 20 ms control pulse at −20 mV. This was followed by a pre-pulse potential between −110 mV and 10 mV for 300 ms, prior to the 20 ms test pulse at −20 mV. Data collection and analysis was performed in pClamp 10 (Molecular Devices) and Excel 2010 (Microsoft), data was fitted with a Boltzmann function in GraphPad Prism (GraphPad Software) to obtain V_1/2_. Data are presented as means ± SEM, where *n* is the number of oocytes used in recordings.

### Graphics

Molecular graphics were rendered with UCSF Chimera 1.10.1^60^.

### Data availability statement

The datasets generated and analysed during the current study are available from the corresponding author on reasonable request.

## Electronic supplementary material

Supplementary Information
