# Supplementary material for: The voltage-gated sodium channel EF-hands form an interaction with the III-IV linker that is disturbed by disease-causing mutations
Source: Sci Rep. 2018 Mar 14;8:4483. doi: 10.1038/s41598-018-22713-y (PMC5852250; doi:10.1038/s41598-018-22713-y)
Supplement: Supplementary file 1 — Supplementary Information [file 41598_2018_22713_MOESM1_ESM.pdf]

The voltage-gated sodium channel EF-hands forms an interaction with the III-IV linker that is disturbed by disease-causing mutations

Bernd R. Gardill[1], Ricardo E. Rivera-Acevedo[1,2], Ching-Chieh Tung[1], Mark Okon[1,3], Lawrence McIntosh[1,3], Filip Van Petegem[1,*]

[1] The University of British Columbia, Department of Biochemistry and Molecular Biology, 2350 Health Sciences Mall, Vancouver, BC Canada V6T 1Z3

[2] Current affiliation: The University of British Columbia, Department of Anesthesiology, Pharmacology, and Therapeutics, 2176 Health Sciences Mall, Vancouver, BC Canada V6T 1Z3

[3] The University of British Columbia, Department of Chemistry, 2350 Health Sciences Mall, Vancouver, BC Canada V6T 1Z3

*Corresponding Author: Filip Van Petegem, The University of British Columbia, Department of Biochemistry and Molecular Biology, 2350 Health Sciences Mall – Rm 2.356, Vancouver, BC Canada V6T 1Z3, Phone: +1 604 827 4267, Email: filip.vanpetegem@gmail.com

SUPPLEMENTARY MATERIAL

Supplemental Figures and Figure legends

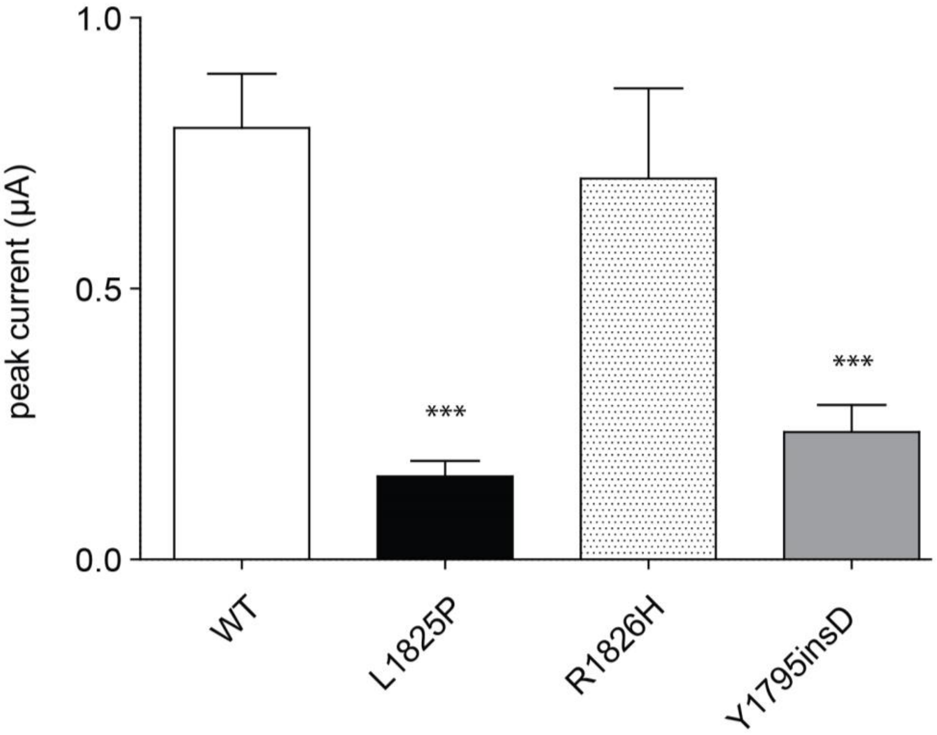

Figure S1 Comparisons of $Na_v1.5$ disease mutants that cause a decrease in current amplitude. All channels were co-expressed with $Na_v\beta1$ subunit in *Xenopus laevis* oocytes. Currents in response to a pulse to -20mV from a -110 mV holding potential and measured 50h after mRNA injection. WT (1.083 ± 0.151 μA *n* = 6); L1825P (0.270 ± 0.57 μA *n* = 7); R1826H (0.884 ± 0.205 μA *n* = 5); Y1795insD (0.283 ± 0.052 μA *n* = 6). Bar graphs represent mean of peak current amplitude (μA) and error bars represent standard error of mean. One-way ANOVA with Dunnetts multiple comparison post test performed to compare WT vs mutant channels mean current amplitude. *** p < 0.001.

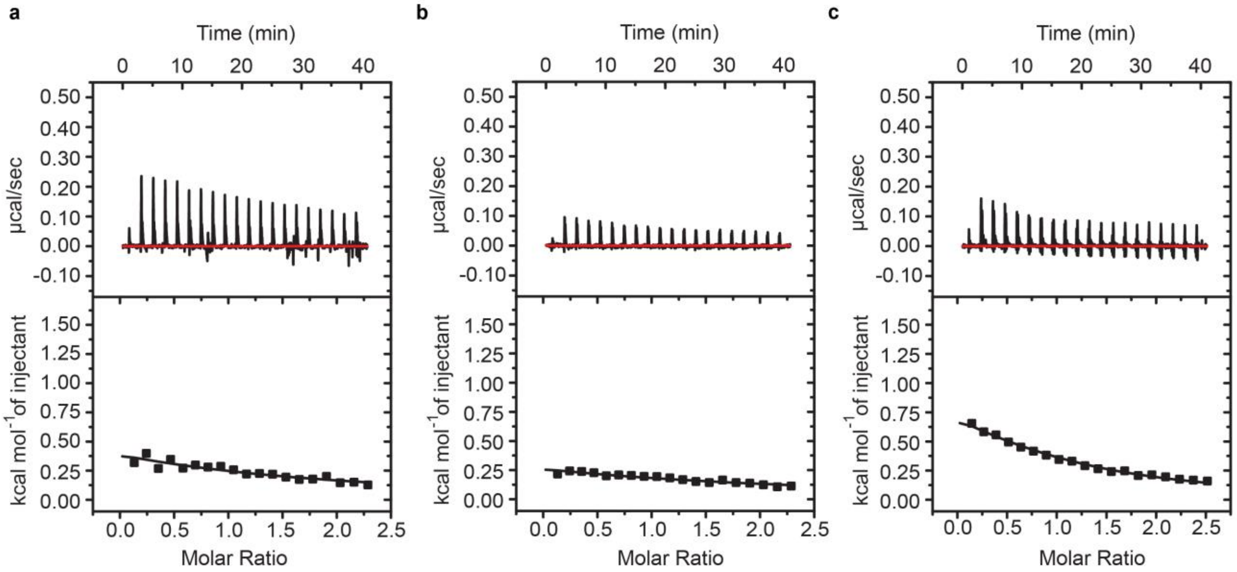

Figure S2 Isothermal titration calorimetry data of EF-hand interaction with designed III-IV linker mutants. EF-hand domain (175 µM) was either titrated with 2 mM mutant peptide or 2.2 mM in the case of R1512E/K1516E. The mutations show a reduction in binding affinity making a reliable fit difficult. The introduced mutations were a) K1492E/K1493E, b) K1499E/K1500E and c) R1512E/K1516E.

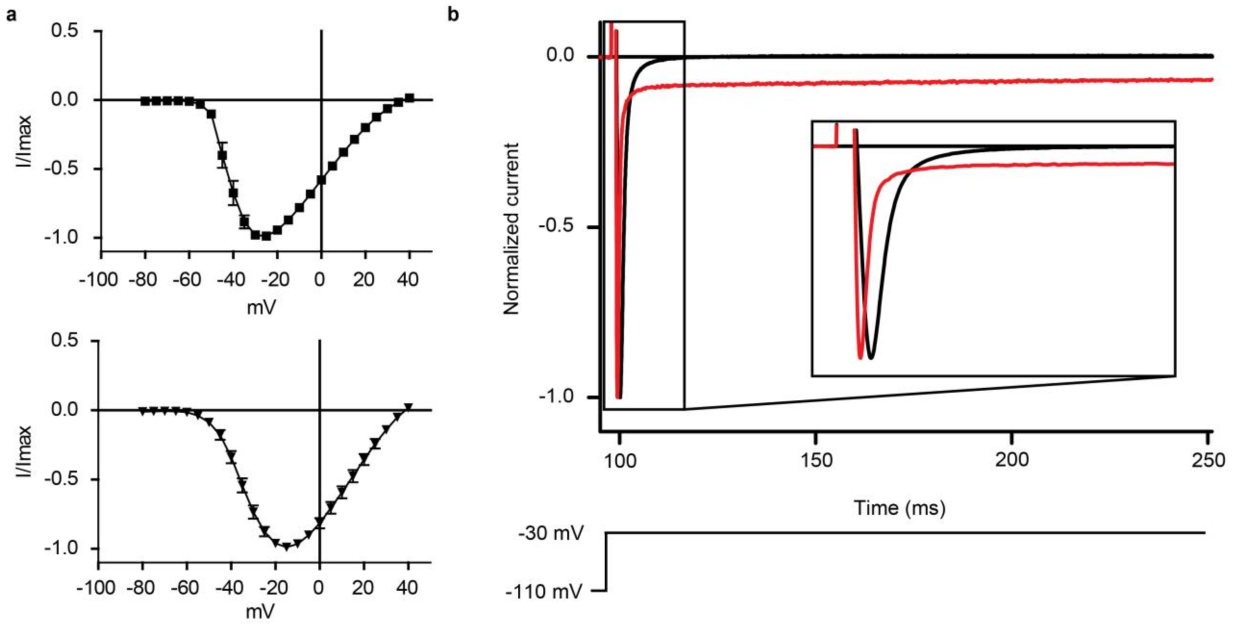

Figure S3 Electrophysiology data obtained for wild-type and K1504/1505E mutant. a) I/V curve for wild-type and mutant, top panel and bottom panel, respectively. A small depolarizing shift is visible for the mutant, possibly through changing the charges near domain IV VSD. b) Normalized single pulse to -30 mV shown for wild-type (black) and mutant (red). The mutant displays a pronounced late current.

Supplemental Tables

Table S1 Thermal denaturation determined by Thermofluor assay

	Denaturation midpoint T_m (°C)	Std. Error of Boltzmann fit	Number of repeats
WT	56.46	0.04528	4
D1790G	50.44	0.09533	6
Y1795C	56.69	0.05187	5
R1826H	41.91	0.02340	6
Q1832E	55.71	0.05585	5
D1839G	57.09	0.03409	5
R1860Gfs*12	42.60	0.2684	3
V1861I	52.35	0.1068	5
K1872N	53.34	0.03216	5

Table S2 Inactivation properties of $Na_v1.5$ co-expressed with β1

	$V_{1/2}$	*k*	*n*
L1825P	-82.59 ± 0.42 mV*	4.833 ± 0.11	7
R1826H	-73.67 ± 0.15 mV	4.587 ± 0.13	13
Y1795insD	-82.25 ± 0.98 mV*	4.643 ± 0.24	8
Wild-type	-73.13 ± 0.07 mV	4.582 ± 0.06	9
K1504E K1505E	-92.61 ± 0.37 mV*	6.259 ± 0.27	10

$V_{1/2}$ is the membrane voltage of half-maximal inactivation and *k* is the slope factor. Errors represented as standard error of the mean (SEM). Number of measured oocytes *n*. * $p<0.0001$ compared to Wild-type

Table S3 Effects and phenotypes of disease mutations

Mutation (reference)	Activation shift (mV)	Inactivation shift (mV)	Late current, increase over WT	Peak current	Clinical phenotype
L1786Q[62]	+15.2	-21.3	3-fold	Decreased	Concealed BrS + LQTS
S1787N[35]	n.s.d.	n.s.d.	2.1- to 2.9-fold, pH and isoform dependent	n.s.d.	BrS + LQTS + SIDS
D1790G[24,25]	n.s.d.	-16, in presence of β1; n.s.d. without β1	n.s.d.	n.s.d.	BrS + LQTS
Y1795insD[30,31]	n.s.d. [31]<br>+8.1[30]	-9.7 [31]<br>-7.3 [30]	3.4-fold [31]<br>n.s.d. [30]	Decreased [30]	BrS + LQTS
Y1795C[36]	n.s.d.	n.s.d.	4 to 5-fold	Increased	LQTS
Y1795H[36]	n.s.d.	-10.5	2.5-fold	Decreased	BrS
L1825P[29,46]	+8.9 [29]<br>n.s.d.[46]	-11[29]<br>-7.3[46]	8-fold[29]<br>2.5-fold[46]	Decreased [29,46]	BrS + LQTS
C1850S[37]	n.s.d.	-11.6	n.a.	Decreased	BrS

SIDS, sudden infant death syndrome; n.s.d. no significant difference; n.a. not available
